# Dendrobium Officinale Polysaccharide Attenuates Insulin Resistance and Abnormal Lipid Metabolism in Obese Mice

**DOI:** 10.3389/fphar.2021.659626

**Published:** 2021-06-14

**Authors:** Jian Qu, Shengyu Tan, Xinyan Xie, Wenqiang Wu, Haihong Zhu, Hang Li, Xiaobo Liao, Jiaojiao Wang, Zhi-Ang Zhou, Song Huang, Qiong Lu

**Affiliations:** ^1^ Department of Pharmacy, The Second Xiangya Hospital, Central South University, Changsha, China; ^2^ Department of Geriatrics, The Second Xiangya Hospital of Central South University, Changsha, China; ^3^ Mathematical Engineering Academy of Chinese Medicine, Guangzhou University of Chinese Medicine, Guangzhou, China; ^4^ Department of Cardiovascular Surgery, The Second Xiangya Hospital, Central South University, Changsha, China

**Keywords:** abnormal lipid metabolism, Dendrobium officinale polysaccharide, insulin resistance, obesity, peroxisome proliferator-activated receptor-γ

## Abstract

**Objectives:** Dendrobium officinale polysaccharide (DOP) is the main active ingredient in a valuable traditional Chinese medicine, which exerts several pharmacological activities including hepatoprotection and hypoglycemic effects. However, the effects of DOP on obesity-associated insulin resistance (IR) and lipid metabolism remain unknown. This study aimed to investigate the role of DOP in IR and abnormal lipid metabolism in obese mice.

**Methods:** IR models were established using 3T3-L1 adipocytes, C2C12 myocytes, and primary cultured hepatocytes exposed to palmitate acid. After treatment with DOP, insulin-stimulated glucose uptake, glucose release, and AKT phosphorylation was detected. Fasting blood glucose, fasting serum insulin, the glucose tolerance test (GTT), and the insulin tolerance test (ITT) were measured to evaluate IR of obese mice. Lipid analysis was conducted to evaluate the effects of DOP on lipid metabolism in obese mice.

**Results:** *In vitro*, DOP treatment ameliorated palmitic acid-induced IR in adipocytes, myocytes, and hepatocytes. DOP regulated cellular insulin sensitivity *via* the peroxisome proliferator-activated receptor-γ (PPAR-γ). Furthermore, administration of DOP significantly reduced the IR and visceral adipose tissue (VAT) inflammation of diet-induced obese (DIO) and the genetically-induced obesity mice (ob/ob) mouse models. In addition, DOP treatment attenuated the high-fat diet (HFD)-induced liver lipid accumulation by reducing liver triglycerides (TG), plasma free fatty acid (FFA), serum cholesterol (TC), and low-density lipoprotein cholesterol (LDL-C) levels, while increasing HDL-C levels.

**Conclusion:** DOP could improve obesity-associated IR and abnormal lipid metabolism through its activities on PPAR-γ, and may serve as a potential therapeutic agent for obesity-associated insulin resistance and lipid metabolism disorder.

## Introduction

With rapid economic growth, obesity is becoming an increasing health concern worldwide. Understandably, obesity was defined as a disease by the Obesity Society (TOS) in 2008 (Belkina and Denis, 2010; Jastreboff et al., 2019). Obesity is characterized by increased circulating fatty acid levels resulting from excessive lipolysis and absorption of basal adipose tissue, which in turn promote the development of ectopic lipid deposition and insulin resistance (IR) (Crowe et al., 2009). Consistently, studies have shown that obesity is a risk factor for metabolic syndrome, which includes IR and dyslipidemia, impaired fasting blood glucose, and hyperinsulinemia (Laaksonen et al., 2002; Meigs et al., 2006). In addition, obesity increases the risk and mortality of many diseases Council of the Obesity (2008), González-Domínguez et al. (2020), especially type 2 diabetes, cardiovascular disease, hypertension, and dyslipidemia (De Lorenzo et al., 2019).

IR refers to the reduction of insulin sensitivity and response to insulin target organs and tissues (liver, adipose tissue, skeletal muscle) due to varied reasons (Samuel and Shulman, 2012). This leads to a decrease in glucose uptake and utilization. Therefore, the body will compensatively secrete excess insulin to maintain the stability of blood sugar, leading to hyperinsulinemia, which in turn leads to dyslipidemia. Dyslipidemia manifests as increased total cholesterol (TC), triglycerides (TG), and free fatty acids (FFA), normal or slightly increased low-density lipoprotein cholesterol (LDL-C), and decreased high-density lipoprotein cholesterol (HDL-C) (Klop et al., 2013). Moreover, the symptoms of IR are impaired glucose absorption in muscles and increased gluconeogenesis in the liver during fasting and postprandial conditions, leading to hyperglycemia (Moller and Kaufman, 2005). Insulin-sensitive tissues (adipose tissue, heart, and liver) are significantly affected by obesity at biomolecular and functional levels (Barazzoni et al., 2018). Excessive fat accumulation can change the function of adipose organs and leads to obesity-related diseases (Ling and Ronn, 2019). In addition, a close relationship between the mechanisms implicated in lipid metabolic disorders and IR has also been reported (Savage et al., 2007). Further, obesity and IR are closely related to inflammation of metabolic tissues, including the liver, muscle, and adipose tissue (Winer et al., 2016; Wu and Ballantyne, 2017; Ghorpade et al., 2018). Excessive fat accumulation and abnormal lipid metabolism caused by obesity induce the transformation of macrophages from the anti-inflammatory M2 polarized state to the pro-inflammatory M1 polarized state, leading to VAT inflammation, thus promoting the development of obesity-related IR (Xu et al., 2003; Lumeng et al., 2007; Kojta et al., 2020). At the same time, IR further aggravates lipid metabolism disorders, leading to a series of metabolic disorders such as diabetes and cardiovascular diseases. The specific manifestations of visceral adipose tissue (VAT) inflammation are increased levels of pro-inflammatory cytokines such as tumor necrosis factor (TNF)-α and interleukin (IL)-6 Hotamisligil et al. (1993), Kern et al. (2001), and decreased levels of anti-inflammatory cytokines IL-10 and IL-4 in obesity-related IR patients (Esposito et al., 2003). Therefore, the prevention or treatment of IR and lipid metabolism disorders may be an effective way to inhibit obesity-related diseases.

*Dendrobium officinale Kimura et Migo* is a valuable traditional Chinese medicine that has been used to treat diabetes, obesity, rheumatoid arthritis, and many other diseases. It has been reported that *Dendrobium officinale Kimura et Migo* can inhibit the effects of oxidative stress and pro-inflammatory cytokines (Teixeira da Silva et al., 2015). Oxidative stress and inflammation may also lead to obesity-related IR (Xu et al., 2003; Gortan Cappellari et al., 2016). Dendrobium officinale polysaccharide (DOP) is the main active component of *Dendrobium officinale Kimura et Migo*, which exerts pharmacological activities including antioxidation, lowering blood lipids and blood sugar levels, and hepatoprotection (Huang et al., 2016). In type 2 diabetic rats, DOP treatment reduced hepatic lipid metabolism disorders and alleviated symptoms of hepatic lipid accumulation (Yang et al., 2020). These studies indicated that DOP is expected to be a novel therapeutic agent for obesity-related diseases including IR and abnormal lipid metabolism. However, to date, there are almost no available mechanistic studies investigating mechanistic studies on the effects of DOP in IR and abnormal lipid metabolism. Meanwhile, the mechanism of DOP in obesity-related IR and abnormal lipid metabolism is still unclear.

The purpose of this study was to explore the effects and mechanisms of action of DOP on obesity-related IR and lipid metabolism disorders. First, at the cellular level, we measured glucose uptake, glucose output, and Akt phosphorylation in insulin-stimulated hepatocytes, muscle cells, and adipocytes, respectively. The results showed that DOP treatment could improve IR-induced obesity. Subsequently, we conducted a mechanism study and found that DOP mainly acted on peroxisome proliferator-activated receptor-γ (PPAR-γ) signaling to regulate insulin sensitivity in diet-induced obesity (DIO) mice. We evaluated how DOP could improve IR in DIO mice by assessing fasting blood glucose and fasting serum insulin levels, the glucose tolerance test (GTT), the insulin tolerance test (ITT), and the inflammation related indicators of VAT. Furthermore, lipid analysis showed that DOP attenuated the abnormal lipid metabolism in DIO mice. We also validated the therapeutic effects of DOP in the genetically-induced obesity mice (ob/ob mice) model. Overall, we expect that our study can provide a basis for the clinical application of DOP in obesity-related IR and lipid metabolism disorders.

## Materials and Methods

### Animal Models

To establish a diet-induced obese (DIO) mice model, two-month-old male C57BL6 mice were fed a high-fat diet (HFD, 60% fat, 20% protein, and 20% carbohydrate) for 3 months. Ob/ob mice are a classical experimental model of obesity-induced IR as evidenced by a plethora of studies. Ob/ob mice harbor a recessive mutation in leptin and are characterized by hyperphagia, obesity, hyperinsulinemia, insulin-resistance (IR), and hyperglycemia; thus, they are commonly used as a model for studies of diabetes and obesity. Ob/ob mice were purchased from Hunan SJA Laboratory Animal Co., Ltd. (license number: SCXK 2019-0004). All animals were housed under 12-h light/dark cycles and were provided unrestricted access to food and water unless otherwise specified.

**FIGURE 1:**
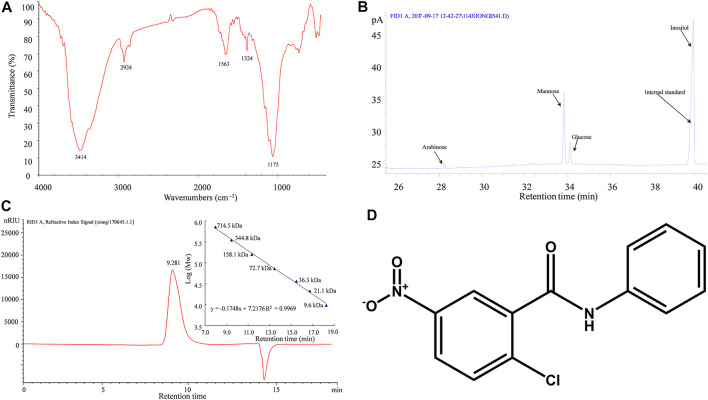
Characterization of DOP **(A)** The FT-IR spectrogram of DOP **(B)** GC chromatogram of monosaccharide composition showed that DOP is allocated to O-H stretching vibrations, C-H stretching vibrations and C-C or C-O stretching vibrations, respectively. These is the characteristic absorption profile of polysaccharides. The monosaccharide composition of DOP was mannose, glucose, and arabinose with a molar ratio of 5.55:1:0.12. **(C)** The average molecular weight of DOP was 393.8 kDa by HPLC profiles of molecular weight measurement. **(D)**The chemical structure diagram of the PPARγ antagonist GW9662.

For *in vivo* DOP treatment, after 3 months of a HFD, the DIO mice were divided into two groups, the control group and the experimental group, the control group received normal saline (NS) orally, and the experimental group was administrated DOP (150 mg/kg) orally, once daily for 3 months. The DOP involved in this study was prepared by the laboratory of Mathematical Engineering Academy of Chinese Medicine, Guangzhou University of Chinese Medicine (Liang et al., 2018; Liang et al., 2019). The proportion of total polysaccharides in the DOP was 93.80%. Moreover, the FT-IR spectrogram showed that DOP has an intense and broad absorption peak around 3414 cm-1, a weak absorption peak at 2924 cm-1 and 1324 cm-1, and an asymmetrical extension at 1175 cm-1 (Figure 1A). And GC chromatogram of monosaccharide composition showed that DOP had the characteristic absorption profile of polysaccharides. DOP is composed of mannose, glucose, and arabinose with a molar ratio of 5.55:1:0.12 (Figure 1B). Meanwhile, the average molecular weight of DOP determined by HPLC profiles of molecular weight measurement was 393.8 kDa (Figure 1C). The structure composition of DOP showed that its formulation was identical to the DOP prepared by Hua (Hua et al., 2004).

All protocols pertaining to animal care and experiments were reviewed and approved by the Institutional Animal Care and Use Committee of the Laboratory Animal Research Center at Xiangya Medical School of Central South University, China.

### Cell Culture and Treatment

Primary hepatocytes, C2C12 myoblasts, and 3T3-L1 preadipocytes were cultured in a humidified incubator at 37°C and supplemented with 5% CO2. Standard protocols were utilized to induce differentiation of these cells. To establish IR models, primary hepatocytes, C2C12, myoblasts and 3T3-L1 preadipocytes were treated with 0.5 mmol/L palmitate acid (PA; Sigma) for 24 h. Glucose levels in the medium were measured to determine whether the IR model was successful. For *in vitro* DOP treatment, 200 μg/ml DOP was added to the culture medium for 48 h.

### Cell Viability

The Cell Counting Kit-8 (CCK-8) was used to assess the viability of 3T3-L1 adipocytes after DOP treatment, as per the manufacturer’s protocol. Absorbance was measured at 450 nm *via* a microplate reader (Thermo Electron Corp).

### Glucose Uptake and Glucose Output Assay

The glucose uptake and glucose output assays were performed as described previously (Su et al., 2019). The medium glucose levels were measured by a Glucose Assay Kit (Abcam, ab65333) according to the manufacturer’s instructions.

### Western Blot and qRT-PCR Analysis

Western blotting analysis was conducted as previously described (Li et al., 2009). Primary antibodies p-AKT (CST9272s; Dilution 1:1,000) and AKT (CST9272s; Dilution 1:1,000) were purchased from Cell Signaling Technology, and PPAR-γ (ab209350, Dilution 1:1,000) was purchased from Abcam. All primary antibodies were incubated at 4°C overnight, the secondary antibodies were diluted to 1:5,000, and specific proteins were visualized by ECL Plus. For qRT-PCR analysis, the total RNA from cultured cells were isolated by TRIzol (Thermo Fisher Scientific). The list of primers used for real-time PCR analysis are described in Table 1.

**TABLE 1:** Primer sequences used for real-time PCR analysis.

Gene	Forward primer	Reverse primer
Actin (mouse)	GGC​TGT​ATT​CCC​CTC​CAT​CG	CCA​GTT​GGT​AAC​AAT​GCC​ATG​T
PPAR-γ (mouse)	GGA​AAG​ACA​ACG​GAC​AAA​TCA​C	TAC​GGA​TCG​AAA​CTG​GCA​C
TNFα (mouse)	TAT​GGC​TCA​GGG​TCC​AAC​TC	CTC​CCT​TTG​CAG​AAC​TCA​GG
IL-6	AGT​TGC​CTT​CTT​GGG​ACT​GA	CAG​AAT​TGC​CAT​TGC​ACA​AC
IL-10	GCC​CTT​CCT​ATG​TGT​GGT​TTG	TTG​AGT​TTC​CGT​ACT​GTT​TGA​GG
IL-4	CCC​CAG​CTA​GTT​GTC​ATC​CTG	CAA​GTG​ATT​TTT​GTC​GCA​TCC​G

### Blood Glucose, Serum Insulin, Insulin Tolerance Test, Glucose Tolerance Test, and HOMA-IR Index

The measurement of blood glucose levels and serum insulin, and the performance of the ITT and GTT, have been reported previously (Ying et al., 2017; Xiao et al., 2020). The following formula was used to calculate the homeostasis model assessment of IR (HOMA-IR) index: [fasting blood glucose levels (mmol/L)] × [fasting serum insulin levels (μU/ml)]/22.5 (Su et al., 2019).

### Lipid Analysis

Approximately, 0.1 g liver was homogenized in phosphate-buffered saline (PBS) solution (1:9 ratio). The mixture was centrifuged at 2,500 rpm for 10 min at 4°C after which the supernatant was collected. The cytoplasm of L02 cells was collected with 2% Triton X-100. Hepatic and hepatocellular TG were quantified using chemical reagent kits from Nanjing Jiancheng Bioengineering Institute (Nanjing, China). In addition, serum lipids including TC, TG, LDL-C, and HDL-C levels were determined using kits from Nanjing Jiancheng Bioengineering Institute (Nanjing, China). Plasma-free fatty acid (FFA) levels were measured enzymatically using a kit from WAKO Chemicals. Absorbance values of all samples were measured using a spectrophotometric system.

### Hematoxylin and Eosin Staining and Oil Red O Staining

Livers samples (4–5 micron in thickness) were placed in cassettes and submerged in 10% formalin solution overnight. Samples were processed in a dehydrating ethanol gradient, followed by xylene incubation and paraffin embedding. Serial 8-μm sections were used for hematoxylin and eosin (H&E) staining and Oil Red O staining.

### Quantification and Statistical Analysis

All data are presented as mean ± SD. For comparisons between two groups, a two-tailed Student’s t-test was used. Comparisons between multiple groups were performed using ANOVA followed by Bonferroni’s post-hoc correction. A *p-*value < 0.05 was considered statistically significant.

## Results

### DOP Ameliorated Cellular Insulin Resistance *In Vitro*

**FIGURE 2:**
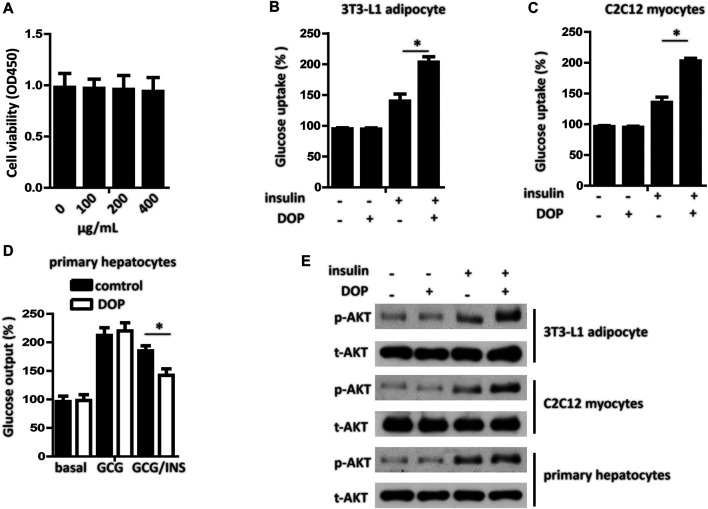
DOP ameliorated cellular insulin resistance *in vitro.* **(A)** 3T3-L1 adipocytes were seeded into 96-well plates at a density of 8 × 103 cells/well and were treated with different concentrations of DOP for 48 h. Cell viability was determined using CCK-8 assay. **(B–D)** Influence of DOP on glucose uptake of 3T3-L1 adipocytes and C2C12 myocytes as well as glucose output of primary cultured hepatocytes **(E)** Insulin stimulated AKT phosphorylation in 3T3-L1 adipocytes, C2C12 myocytes, and primary cultured hepatocytes. Data are presented as mean ± SD. Statistical significance was determined using analysis of variance (ANOVA) for A, and Student’s t-test for B, C, and D.* means *p* < 0.05.

The cytotoxicity of DOP was assessed by the CCK-8 assay, which revealed DOP did not affect cell viability at concentrations of 100, 200, and 400 μg/ml (Figure 2A). To explore whether DOP participated in the regulation of insulin sensitivity, we treated PA-induced adipocytes, myocytes, and hepatocytes with DOP. As expected, DOP treatment significantly promoted the insulin-stimulated glucose uptake of 3T3-L1 adipocytes and C2C12 myocytes (Figures 2B,C), while the glucose output of primary cultured hepatocytes decreased (Figure 2D). In addition, the insulin-stimulated AKT phosphorylation of 3T3-L1 adipocytes, C2C12 myocytes, and primary cultured hepatocytes was increased following DOP treatment (Figure 2E). Taken together, these results suggested that DOP ameliorated cellular IR of adipocytes, myocytes, and hepatocytes *in vitro*.

### DOP Regulated Cellular Insulin Sensitivity by Activating PPAR-γ

**FIGURE 3:**
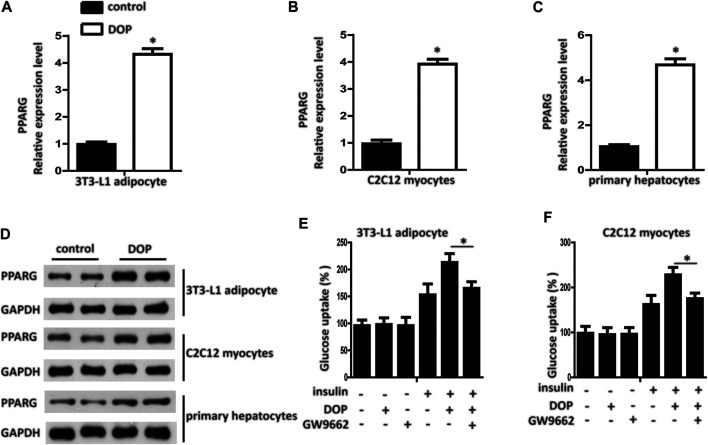
DOP regulated cellular insulin sensitivity by activating PPAR-γ **(A–C)** Expression levels of PPAR-γ in 3T3-L1 adipocytes, C2C12 myocytes, and primary cultured hepatocytes treated with DOP (200 μg/ml, 48 h) **(D)** Western blot analysis of the protein levels of PPAR-γ of 3T3-L1 adipocytes, C2C12 myocytes, and primary cultured hepatocytes treated with DOP. **(E–F)** Effects of GW9662 (10 μM, 24 h) on insulin-stimulated glucose uptake of 3T3-L1 adipocytes and C2C12 myocytes. Data are presented as mean ± SD. Statistical significance was calculated by two-tailed Student’s t-test. * means *p* < 0.05.

Pharmacological activation of PPAR-γ has emerged as an effective method for treating diabetes. We found that the transcript levels of PPAR-γ mRNA in 3T3-L1 adipocytes, C2C12 myocytes, and primary cultured hepatocytes treated with DOP were elevated (Figures 3A–C). Western blotting analysis further confirmed that DOP could promote the protein expression levels of PPAR-γ (Figure 3D). These observations suggested that DOP increased the expression of PPAR-γ.

To validate whether DOP regulated cellular insulin sensitivity through the activation of PPAR-γ, we treated PA-induced adipocytes and myocytes with GW9662, a PPAR-γ antagonist. GW9662 is a potent and selective PPAR-γ antagonist with an IC50 of 3.3 nM, showing 10 and 1000-fold selectivity over PPARα and PPAR-δ, respectively. The chemical structure is shown in Figure 1D. The effects of DOP on 3T3-L1 adipocytes and C2C12 myocytes was abrogated by GW9662, which indicated that DOP failed to improve the insulin sensitivity if PPAR-γ was inhibited (Figures 3E,F). Taken together, these results indicated that DOP regulated cellular insulin sensitivity by activating PPAR-γ.

### Administration of DOP Reduced the Insulin Resistance of Obese Mice

**FIGURE 4:**
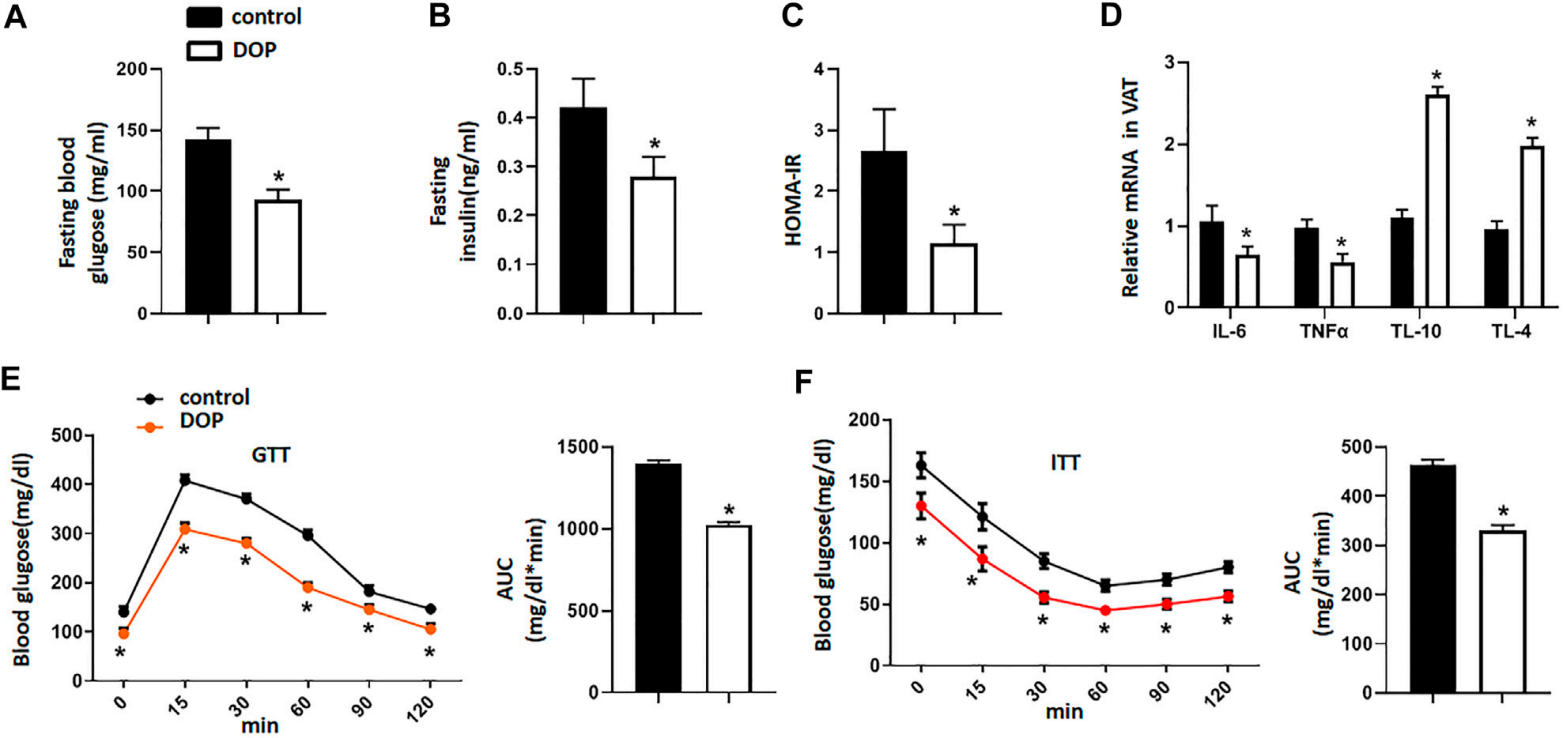
Administration of DOP reduced insulin resistance in obese mice **(A–C)** The fasting blood glucose levels, fasting serum insulin, and HOMA-IR index of obese mice administered NS or DOP **(D)** Inflammation-related mRNA levels of VAT in obese mice treated with NS or DOP **(E–F)**. The GTTs and ITTs in obese mice treated with NS or DOP (*n* = 5 per group). AUC: area under curve. Data are presented as mean ± SD. Statistical significance was calculated using the two-tailed Student’s t-test. * means *p* < 0.05.

To explore the therapeutic potential of DOP on the IR of obese mice, we treated obese mice with normal saline (NS) or DOP. Notably, mice treated with DOP had lower fasting blood glucose levels and fasting serum insulin (Figures 4A,B). The HOMA-IR index was also lowered in the DOP-treated group (Figure 4C). Moreover, DOP could also suppress VAT inflammation. The levels of pro-inflammatory cytokines IL-6 and TNFα were also decreased, while the anti-inflammatory cytokines IL-10 and IL-4 were increased (Figure 4D). Furthermore, the results of the GTT and ITT revealed that glucose tolerance and clearance in mice were increased after administration of DOP (Figures 4E,F). Together, these findings suggested that administration of DOP reduced IR and adipose tissue inflammation in obese mice.

### DOP Impaired Lipid Metabolism Disorder in Obese Mice

**FIGURE 5:**
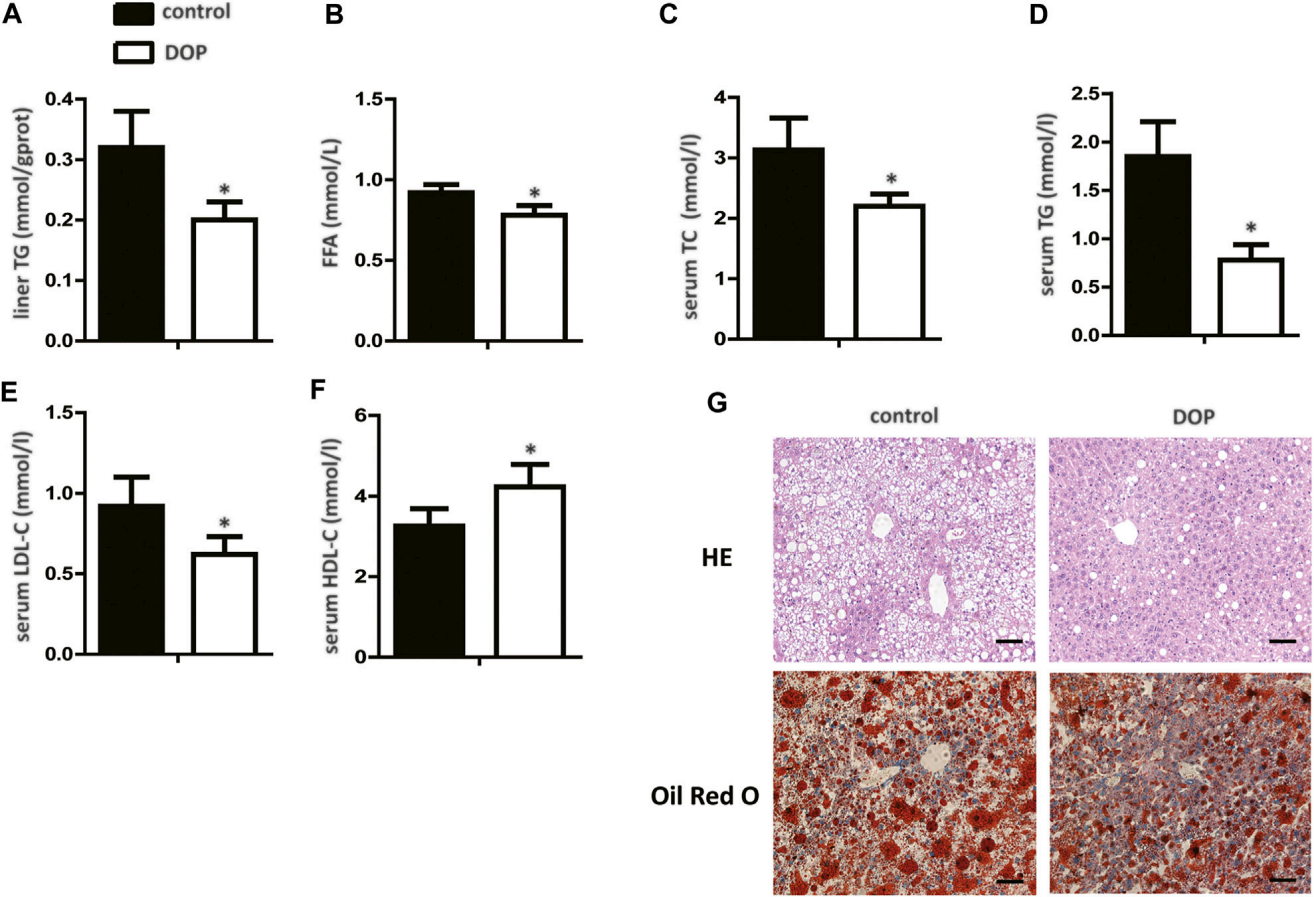
DOP impaired lipid metabolism disorder in obese mice **(A)** TG levels in the liver after treatment with DOP. **(B–F)** The serum levels of TC, TG, LDL-C, and HDL-C after treatment with DOP (*n* = 5 per group) **(G)** Representative images of H&E staining (G, top) and Oil Red O staining (G, bottom). Scale bars: 300 μm. Data are presented as mean ± SD. Statistical significance was calculated using the two-tailed Student’s t-test. * means *p* < 0.05.

To further explore the effect of DOP on lipid metabolism in obese mice, serum, and hepatic lipid levels were assayed. DOP-treated mice exhibited significantly lower TG levels in the liver when compared with untreated mice (Figure 5A). In addition, FFA, serum TC, TG, and LDL-C levels in DOP-treated mice were also lower than in the control group (Figures 5B–E). Importantly, DOP treatment was effective in increasing HDL-C levels (Figure 5F). The H&E staining and Oil Red O staining of the liver samples indicated that compared with the control group, the lipid levels and volumes were decreased in DOP treatment group in ob/ob mice (Figure 5G). Taken together, these results showed that DOP could ameliorate lipid metabolism disorders in obese mice.

### DOP Treatment Alleviated Insulin Resistance in Ob/Ob Mice

**FIGURE 6:**
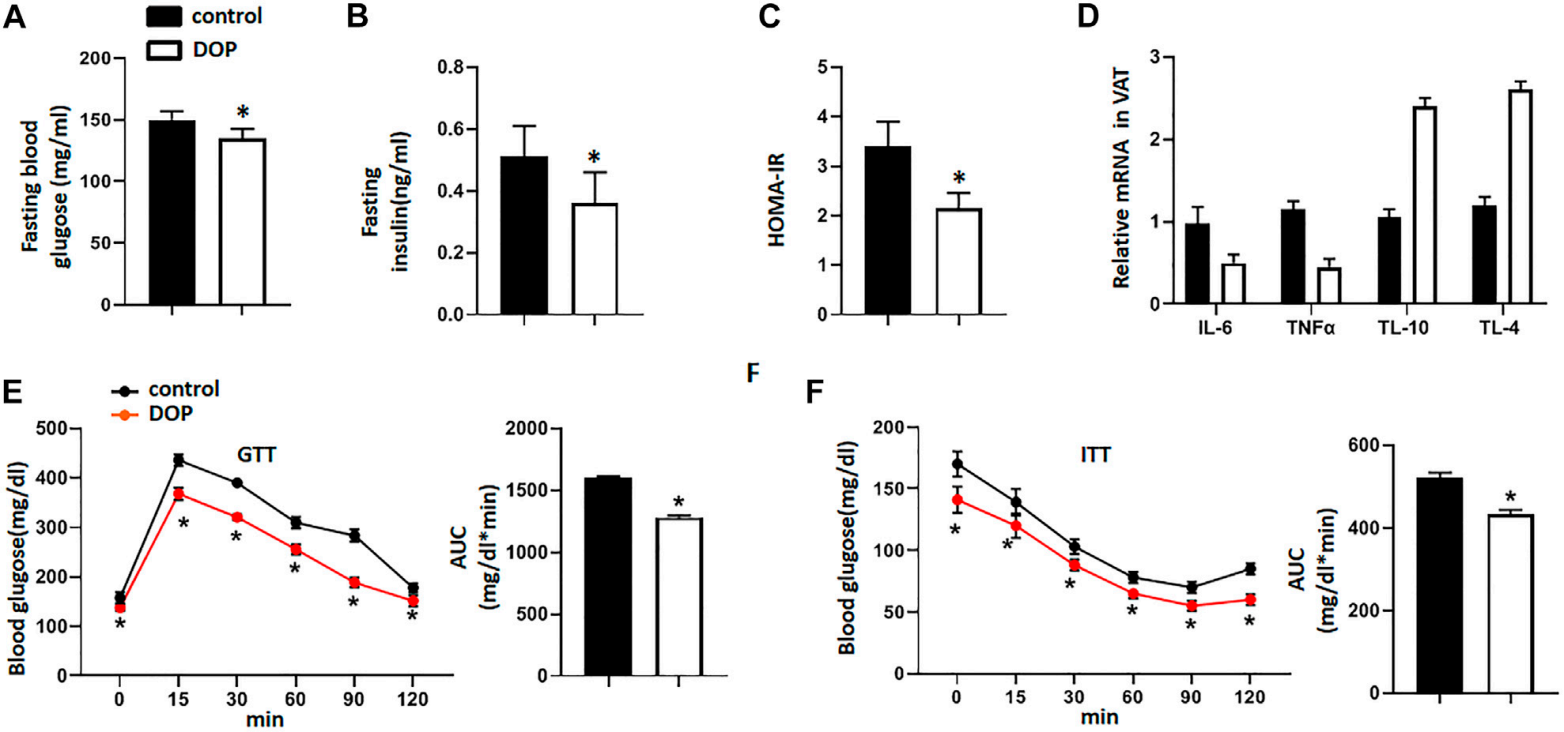
DOP treatment alleviated insulin resistance in ob/obmice **(A–C)** The fasting blood glucose levels, fasting serum insulin, and HOMA-IR index of ob/ob mice administered NS or DOP **(D)** Inflammation-related mRNA levels of VAT in ob/ob mice treated with NS or DOP **(E–F)**. The GTTs and ITTs in ob/ob mice treated with NS or DOP (*n* = 5 per group). AUC: area under curve. Data are presented as mean ± SD. Statistical significance was calculated using the two-tailed Student’s t-test. * means *p* < 0.05.

To further confirm the therapeutic effect of DOP, ob/ob mice were orally administered DOP as mentioned above. Similar to the observations in obese mice, ob/ob mice treated with DOP also exhibited decreased fasting blood glucose levels, fasting serum insulins, and a lower HOMA-IR index (Figures 6A–C). In addition, VAT inflammation was also restrained by DOP treatments exhibited by reduced levels of inflammatory cytokines (IL-6 and TNFα) and elevated expression of anti-inflammatory cytokines (IL-10 and IL-4) (Figure 6D). The results from the GTT and ITT indicated that the application of DOP could improve the glucose intolerance of ob/ob mice (Figures 6E,F). Thus, all these results indicated that DOP treatment could alleviate IR and adipose tissue inflammation in ob/ob mice.

## Discussion

Obesity is the basis of IR, which is a potential cause of a complex metabolic syndrome, including hypertension, elevated fasting blood glucose, low HDLC, and elevated TG levels (Beale, 2013). Therefore, there is an urgent need to identify potential therapeutic agents and suitable targets to attenuate IR and abnormal lipid metabolism in obese patients. In this study, we confirmed the potential therapeutic target of PPAR-γ in obese mice and the role of DOP in attenuating IR and abnormal lipid metabolism.

IR is defined as the decreased sensitivity to insulin and response of insulin to target organs and tissues (liver, adipose tissue, skeletal muscle) (Samuel and Shulman, 2012). IR can lead to an increase in intracellular glucose concentration and a decrease in glucose uptake. However, for the liver, IR is characterized by an inability to inhibit liver glucose production and glycogen decomposition (Randle et al., 1963; Randle et al., 1964; Randle et al., 1965). AKT protein kinase in adipocytes, muscle cells, and liver cells is the key kinase regulating glucose homeostasis (Czech, 2017). Previous studies have found that fatty acid stimulation is an early event of IR (Lee et al., 2014). In this study, we used PA to treat adipocytes, myocytes, and hepatocytes to establish IR models of the three cell types. We then measured glucose uptake or output and insulin-stimulated AKT phosphorylation in these insulin-resistant cells with or without DOP treatment. As expected, DOP treatment significantly promoted the insulin-stimulated glucose uptake of 3T3-L1 adipocytes and C2C12 myocytes, while the glucose output from hepatocytes decreased. Therefore, it is likely that DOP is an agent for potential treatment or prevention of IR.

Multiple mechanisms are involved in the IR of type 2 diabetes. PPAR-γ is a subfamily of nuclear receptors. Activation of PPAR-γ has been reported to improve blood glucose control and systemic insulin sensitivity in patients with type 2 diabetes (Semple et al., 2006). The activation of nuclear receptor PPAR-γ in adipose tissue, liver, and muscle is a determinants of insulin sensitivity (Lu et al., 2011; Germoush et al., 2019). In accordance with previous studies, our results showed that DOP could up-regulate the expression of PPAR-γ in adipocytes, myocytes, and hepatocytes.

IR can lead to hyperinsulinemia and impaired fasting blood glucose (elevated fasting blood glucose). Further, ITT, and GTT are also effective methods to evaluate IR (Guerre-Millo et al., 2001). IR can be diagnosed clinically by a homeostasis model of IR (HOMA-IR) based on fasting blood glucose and fasting serum insulin levels (Yee et al., 2019). To further explore the therapeutic effects of DOP on IR, we conducted *in vivo* experiments using and obese IR mouse model. The results showed that the levels of fasting blood glucose and fasting serum insulin in the DOP-treated group were significantly lower than those in the control group. Through the ITT and GTT, we found that the blood glucose concentration of mice treated with DOP decreased significantly, which indicated that DOP treatment could significantly increase the glucose tolerance of obese mice and clearance rate of insulin to blood glucose. Furthermore, an increase in obesity-induced lipid storage leads to adipose tissue dysfunction and promotes adipocyte secretion of pro-inflammatory cytokines, including TNF-α and IL-6. Moreover, due to the transformation of M2 macrophages to M1 in obesity tissues, the expression of anti-inflammatory cytokines IL-10 and IL-4 secreted by M2 macrophages is decreased (Taylor, 2021). Changes in the expression of these markers lead to VAT inflammation, which in turn promotes IR. However, our study showed that DOP treatment can reverse the changes in pro-inflammatory and anti-inflammatory cytokines caused by obesity. These results once again confirmed the therapeutic effects of DOP on obesity-induced IR.

Typical obesity-induced dyslipidemia includes elevation in TC, TG, and FFA levels; normal or mildly elevated LDL-C; and decreased HDL-C levels (Klop et al., 2013). Multiple studies have shown that these changes in lipid metabolism are closely related to IR (Reaven et al., 1988; Frayn, 1993; Boden et al., 1994; Ormazabal et al., 2018). Therefore, we analyzed the levels of serum and liver lipids in obese mice with or without DOP treatment. The FFA and serum TC, TG, and LDL-C levels were lower and the serum HDL-C levels were higher in DOP-treated mice than control mice. These results were similar to the study by Yang et al. study, whereby DOP treatment reduced the metabolic disorder of liver lipids (fatty acids, TG, and glycerolipids) and reduced the symptoms of lipid accumulation in the liver of type 2 diabetic rats (Yang et al., 2020). Thus, we believe that DOP treatment can improve disorders of obesity-induced lipid metabolism.

Interestingly, only a handful of studies have been published in areas related to DOP and obesity to date. The present study investigated whether DOP could improve IR and lipid metabolism disorders in DIO mice. In order to better demonstrate the therapeutic effects of DOP, we used the ob/ob mouse, a genetic model of obesity. Leptin-deficient (ob/ob) mice are an excellent model of obesity and IR Tomita et al. (1992) and are characterized by elevated insulin and glucose levels and by elevated plasma TG and TC, dyslipidemia, and IR (Dubuc, 1976; Fellmann et al., 2013). Concordant with DIO mice, DOP treatment significantly improved glucose tolerance and insulin clearance of blood glucose in ob/ob mice. Remarkably, DOP has also been shown to inhibit VAT inflammation and liver lipid deposition in the ob/ob mouse model of hereditary obesity. Further, this model was used to further confirm the therapeutic effects of DOP on obesity-related IR and lipid metabolism disorders.

There is a limitation to be noted in our study. Hyperinsulinemic-euglycemic clamp studies are the “gold standard” for evaluating IR, but in our study, we did not adopt this method due to the limitations of the experimental conditions. Nonetheless, an important strength of our study is that it is the first to confirm that DOP can reduce IR and improve abnormal lipid metabolism in obese mice.

In conclusion, DOP can improve insulin sensitivity by up-regulating the expression of PPAR-γ, thus improving obesity-related IR. In addition, DOP can treat disorders of lipid metabolism in obese mice. To our knowledge, our study is the first to confirm that DOP can reduce IR and improve abnormal lipid metabolism in obese mice, which provides a novel therapeutic option for the treatment of obesity-related IR and lipid metabolism disorders.

## Data Availability

The original contributions presented in the study are included in the article/Supplementary Material, further inquiries can be directed to the corresponding authors.

## Abbreviations

DIO, Diet-induced obese; DOP, Dendrobium officinale polysaccharide; FFA, Free fatty acid; GTT, Glucose tolerance test; HDL-C, High-density lipoprotein cholesterol; HFD, High-fat diet; HOMA-IR, Homeostasis model assessment of insulin resistance; IR, Insulin resistance; ITT, Insulin tolerance test; LDL-C, Low-density lipoprotein cholesterol; PPAR-γ, Peroxisome proliferator-activated receptor-γ; TC, Serum cholesterol; TG, Triglycerides; VAT, Visceral adipose tissue.
